# The TPLATE complex mediates membrane bending during plant clathrin–mediated endocytosis

**DOI:** 10.1073/pnas.2113046118

**Published:** 2021-12-14

**Authors:** Alexander Johnson, Dana A. Dahhan, Nataliia Gnyliukh, Walter A. Kaufmann, Vanessa Zheden, Tommaso Costanzo, Pierre Mahou, Mónika Hrtyan, Jie Wang, Juan Aguilera-Servin, Daniël Van Damme, Emmanuel Beaurepaire, Martin Loose, Sebastian Y. Bednarek, Jiří Friml

**Affiliations:** ^a^Institute of Science and Technology, 3400 Klosterneuburg, Austria;; ^b^Department of Biochemistry, Hector F. DeLuca Laboratories, University of Wisconsin–Madison, Madison, WI 53706;; ^c^CNRS, INSERM, Laboratory for Optics and Biosciences Ecole Polytechnique, Institut Polytechnique de Paris, 91128 Palaiseau, France;; ^d^Department of Plant Biotechnology and Bioinformatics, Ghent University, 9052 Ghent, Belgium;; ^e^VIB Center for Plant Systems Biology, 9052 Ghent, Belgium

**Keywords:** clathrin-mediated endocytosis, TPLATE, *Arabidopsis*, membrane remodeling

## Abstract

Clathrin-mediated endocytosis is the major route of entry of cargos into cells and thus underpins many physiological processes. During endocytosis, an area of flat membrane is remodeled by proteins to create a spherical vesicle against intracellular forces. The protein machinery which mediates this membrane bending in plants is unknown. However, it is known that plant endocytosis is actin independent, thus indicating that plants utilize a unique mechanism to mediate membrane bending against high-turgor pressure compared to other model systems. Here, we investigate the TPLATE complex, a plant-specific endocytosis protein complex. It has been thought to function as a classical adaptor functioning underneath the clathrin coat. However, by using biochemical and advanced live microscopy approaches, we found that TPLATE is peripherally associated with clathrin-coated vesicles and localizes at the rim of endocytosis events. As this localization is more fitting to the protein machinery involved in membrane bending during endocytosis, we examined cells in which the TPLATE complex was disrupted and found that the clathrin structures present as flat patches. This suggests a requirement of the TPLATE complex for membrane bending during plant clathrin–mediated endocytosis. Next, we used in vitro biophysical assays to confirm that the TPLATE complex possesses protein domains with intrinsic membrane remodeling activity. These results redefine the role of the TPLATE complex and implicate it as a key component of the evolutionarily distinct plant endocytosis mechanism, which mediates endocytic membrane bending against the high-turgor pressure in plant cells.

## Significance

Endocytosis transports cargos inside the cell by creating spherical vesicles from the plasma membrane. This membrane remodeling requires proteins to generate force to bend the membrane inwards, overcoming the high-turgor pressure in plant cells. However, as plants create clathrin-coated vesicles without actin, the machinery to bend membranes during endocytosis is entirely unknown and appears distinct from other model systems. Here, we refine the physiological role of the plant-specific and essential endocytic TPLATE complex. We find it localizes outside of clathrin-coated vesicles and mediates membrane bending, contrasting with previous predictions. We further demonstrate that the TPLATE complex contains protein domains which have intrinsic membrane-bending activity; thus, we identify a component of the unique endocytosis membrane-bending machinery in plants.

Clathrin-mediated endocytosis (CME) is a critical eukaryotic cellular process that regulates a wide range of physiological processes, for example, mediating the internalization of receptors and transporters (1). During CME, a small area of the plasma membrane (PM) is bent into a “dome” shape with a wide aperture and then is further remodeled into an “omega” shape with a narrow neck and a high degree of curvature (2, 3), all while overcoming opposing intracellular forces like turgor pressure. The mechanisms driving this process in mammalian and yeast systems have been the subject of extensive study for the better part of five decades, which has led to the identification of key proteins that provide the force required to overcome these opposing forces (3, 4). Critically, actin has been established to be essential for membrane bending in systems with high-turgor pressures (3, 5). In stark contrast, plant CME characterization is in its infancy. Indeed, it had been postulated for many years that, because of the extreme levels of turgor pressure in plants, CME was physically impossible in most plant cells (6). However, while it is now well established that CME does occur in planta and plays key developmental and physiological roles (7), the machinery and mechanisms that drive CME against the unique biophysical properties of plant cells are yet to be clearly identified. For example, it had long been thought that plant CME relies on actin to overcome the extreme turgor pressure; however, it has recently been demonstrated that plant CME is independent of actin, highlighting that plants have evolved a distinct solution to bending membranes against high-turgor pressures (8). A further mechanistic divergence of plant CME is manifested by the presence of the octameric TPLATE complex (TPC), in which all eight members share the same localizations and dynamics at sites of plant CME, and is essential for both CME and plant survival (9, 10). While this complex is conserved in some biological systems, for example Dictyostelium, it is notably absent from mammalian and yeast genomes (9, 11, 12). Based on static interaction and localization data, the TPC has been proposed to be a classical endocytosis adaptor protein, chiefly acting to bind cargo in the clathrin-coated vesicle (CCV) and driving the coat assembly and has thus been predicted to localize beneath the clathrin coat (9, 13). Here, we use a range of live imaging methodologies and biochemical analysis of CCVs from plant cells and find that the TPC is localized outside the CME vesicle, contrasting with previous predictions. This peripheral localization of TPLATE suggested a role in membrane bending. By using electron microscopy with cells subjected to TPC disruption, we found only flat clathrin structures, confirming a redefined role for the TPC in mediating membrane bending. Finally, we identify that the plant-specific TPC members, AtEH1/Pan1, contain domains which have intrinsic membrane remodeling activity.

## Results

### TPLATE Is Localized outside of Clathrin-Coated Vesicles.

**Fig. 1.**
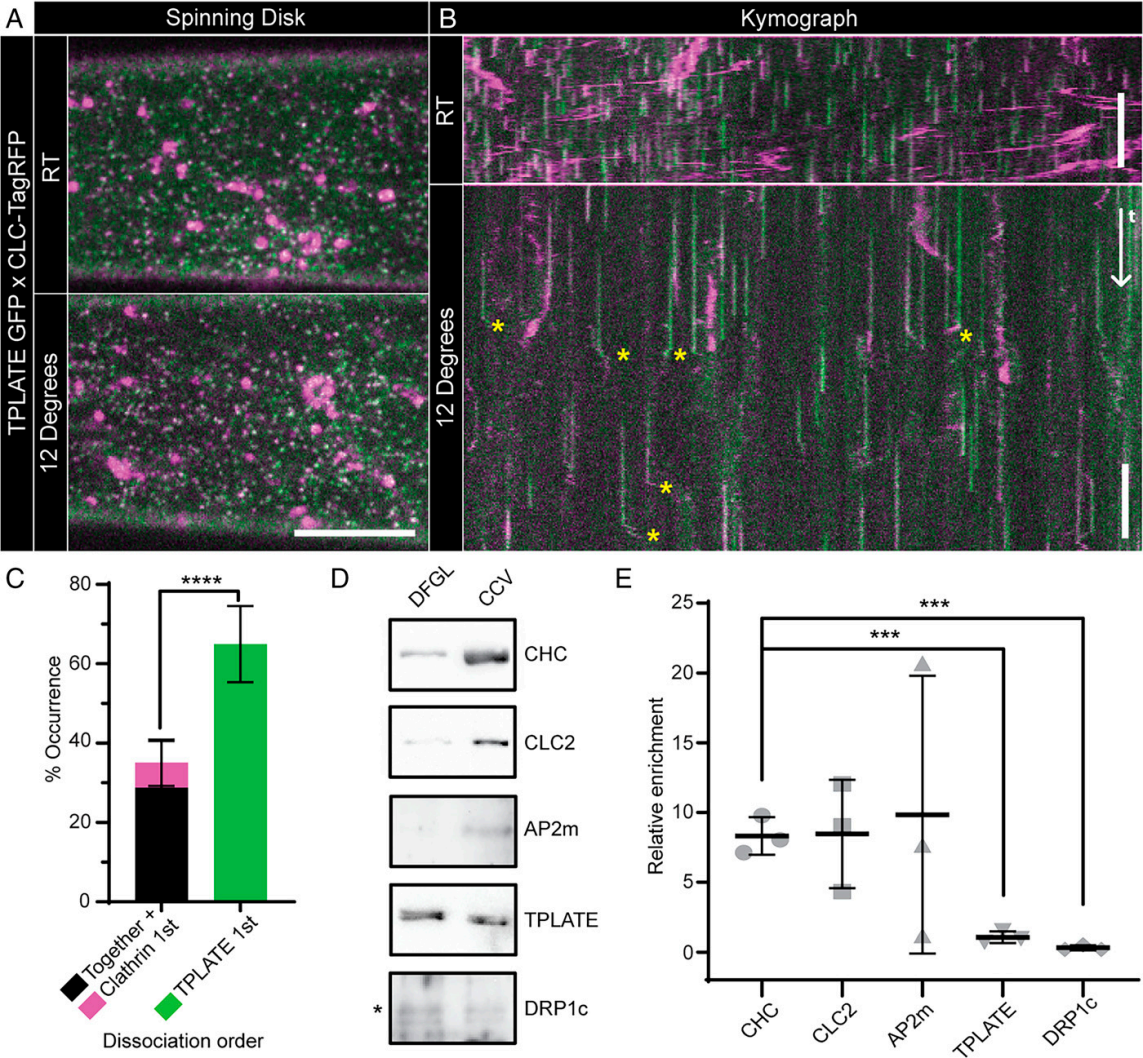
TPLATE is only loosely associated with CCVs. Example spinning-disk images (*A*) and kymographs (*B*) of *Arabidopsis* hypocotyl epidermal cells expressing TPLATE-GFP (green) and CLC2-TagRFP (magenta) at either room temperature (RT) or 12 °C. Yellow asterisks note example departure traces where CCVs are visible after dissociation from the PM. (*C*) Quantification of departure traces based on the order of departure (*SI Appendix*, Fig. S1*B*). *n* = 14 cells from independent plants, 258 departure traces. ****P < 0.0001, Student’s *t* test. Representative Western blots of endocytosis proteins during CCV purification (*D*) and quantification of proteins in the CCV fraction relative to an earlier purification step (DFGL) (*E*). *n* = 3 independent CCV purifications. ***P > 0.001, Student’s *t* tests compared to clathrin heavy chain (CHC). Plots, mean ± SD. (Scale bars, *A*, 5 µm; *B*, 60 s.)

While the TPC has been predicted to localize under the clathrin coat of CCVs, recent total internal reflection fluorescence microscopy (TIRF-M) of CME events in planta suggested that once the endocytic CCV departs from the PM, TPLATE dissociates from the CCV prior to the loss of clathrin (8). This did not support the proposed classical adaptor functions of TPLATE, which, as assumed to be localized under the clathrin coat, should have an equivalent/slower dissociation relative to clathrin. Furthermore, the canonical adaptor AP2 and TPLATE have different dynamics at colocalized foci on the PM (9). To further assess and quantify the dissociation of TPLATE from the CCV, we analyzed CME events marked with clathrin light-chain 2 (CLC2-tagRFP) and TPLATE-GFP obtained using a spinning-disk microscope equipped with a sample-cooling stage (10) (Fig. 1*A*). As this imaging modality provides an increase in the illumination volume of the cytoplasm compared to TIRF-M, and cooling the sample slowed the dynamics of cellular processes (10), it allowed a more precise visualization of the dissociation sequence of proteins from the CCV once it is freed from the PM (*SI Appendix*, Fig. S1*A*). This imaging approach resulted in kymographs of individual CME events with a visible lateral divergence of fluorescence signals at the end of the CME events (Fig. 1*B*), which represent the movement of CCVs once departed from the PM. These “departure traces” were analyzed to establish the sequence of TPLATE and CLC2 dissociation. We found that the significant majority of visible departure traces displayed differential dissociation of these proteins, critically where TPLATE departed the CCV before CLC2 (Fig. 1*C* and *SI Appendix*, Fig. S1*B*). Given that the TPC member proteins are reported to have the same dynamics on the membrane as each other (10), this early departure of TPLATE from CCVs argued against the model that the TPC is entrapped within the clathrin coat of the CCV.

To further query the nature of the association of the TPC with CCVs, we performed a Western blot analysis of purified CCV preparations from plant cells. While we found an enrichment of the clathrin isoforms and the canonical adaptor AP2 in the purified CCV fraction, TPLATE was not enriched (Fig. 1 *D* and *E*). The depletion of TPLATE was observed in parallel with a similar lack of enrichment of dynamin-related protein 1c (DRP1c), which functions outside the CCV (14). Critically, these finding have recently been confirmed by mass spectrometry of purified CCV samples in which none of the TPC members were found to be significantly enriched in CCVs (15). The absence of TPLATE enrichment in purified CCVs demonstrates that TPLATE is not incorporated within CCV structures as previously predicted, and when combined with the early departure of TPLATE from CCVs (Fig. 1*C*), suggests that the TPC is in fact is loosely associated with CCVs outside of the assembled clathrin coat.

**Fig. 2.**
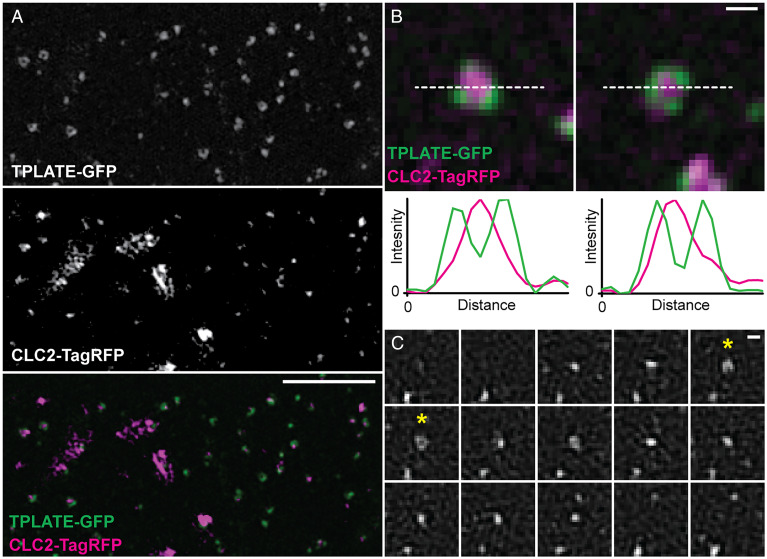
The TPC is localized at the rim of CME events. (*A*) Representative 3D SIM image of an *Arabidopsis* root epidermal cell expressing TPLATE-GFP and CLC2-TagRFP. (*B*) Examples of individual endocytosis structures and line plots (white dotted line) of their fluorescent intensities. (*C*) TIRF-SIM example of TPLATE-GFP dynamics in an *Arabidopsis* root epidermal cell (see Movie S1 for a larger field of view). Asterisks note when a ring structure is formed. Frame interval is 5 s. (Scale bars, *A*, 3 µm; *B* and *C*, 200 nm.)

To precisely determine the localization of TPLATE at CME events, we used three-dimensional (3D) and TIRF structured illumination microscopy (SIM) to examine live plants expressing TPLATE-GFP and CLC2-TagRFP at physiological temperatures. This mode of imaging provides a doubling of the lateral resolution when compared to diffraction limited approaches (16), which had previously been used to probe the localization of TPLATE and CLC. We observed that TPLATE, in addition to presenting as individual foci, often appeared as crescent-shaped or ring structures (Fig. 2*A* and *SI Appendix*, Fig. S2*A*). This was in contrast to CLC2, which was always found as discrete foci on the PM or large structures representing trans-Golgi/early endosomes (8). We found that ∼68% of TPLATE colocalized with CLC2 foci, agreeing with previously published results (9). Upon closer inspection of the colocalized events, we were able to spatially resolve that ∼17% of colocalization events showed that TPLATE formed a ring or crescent around the CLC2 foci, whereas the inverse arrangement was never observed (Fig. 2*B* and *SI Appendix*, Fig. S2*B*), suggesting that TPLATE localizes outside of the clathrin coat assembly during CME. In further support of this, when we examined plants expressing TPLATE-GFP and AP2A1-tagRFP, we found a similar pattern in which ∼18% of colocalized events showed TPLATE surrounding AP2 in ring and crescent patterns (*SI Appendix*, Fig. S2*A*). To gain further insight into the TPLATE ring arrangements, we visualized the dynamics of TPLATE on the PM. In tracks which have a similar lifetime to bona fide CME events [∼45 s (8)], we observed that TPLATE first appeared as a spot and over time forms a ring, which then closed back to a spot before disappearing (Fig. 2*C* and Movie S1). This suggests that at the beginning of the CME event, TPLATE and clathrin are both present in an area below the resolution of SIM [∼100 nm (16)], but as the clathrin-coated invagination grows in diameter to create the dome shape invagination [which plant transmission electron microscopy (TEM) data has shown has a diameter over the SIM resolution limit (2)], TPLATE is excluded from the invaginating CCV and coat formation and is localized at the rim around the CCV. This further supports that TPLATE is localized at the periphery of CCV events rather than within the invaginating endocytic dome within the clathrin coat. In further support of this peripheral TPC localization, we found that other core members of the TPC also formed these ring arrangements on the PM using TIRF-SIM (*SI Appendix*, Fig. S2*C*), confirming that the whole TPC is localized at the periphery of CME events.

### The TPLATE Complex Is Required for Membrane Bending during Clathrin-Mediated Endocytosis.

**Fig. 3.**
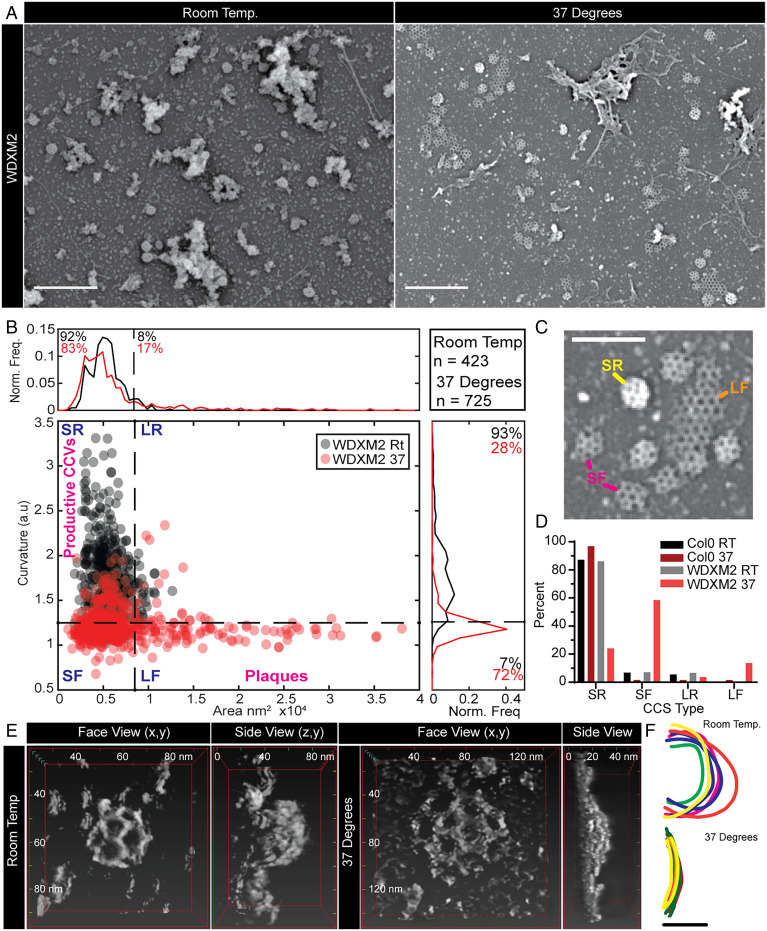
TPC disruption prevents membrane bending during plant CME. (*A*) SEM of metal replicas of unroofed WDXM2 root protoplast cells. (*B*) Scatter plot of the area and curvature of CCSs in WDXM2 cells incubated at room temperature (RT) (gray dots) or 37 °C (red dots) for 4 h. The graph is divided into four sections in order to classify the CCSs based on their shape: SR (small and round), SF (small and flat), LR (large and round), and LF (large and flat, plaques). The small/large threshold is based on an area of 105-nm diameter, and the round/flat threshold was based on measurements made of clathrin plaques observed in TPC disruption conditions (e.g., “LF” example in *C*). (*C*) Example CCSs of these classifications. (*D*) Percentage populations of these classifications in wild-type (Col-0) and WDXM2 cells subjected to RT or 37 °C incubations. Data pooled from multiple experiments; *n* = Col-0 RT, 3 and 588 CCSs; Col-0 37 °C, 4 and 127 CCSs; WDXM2 RT, 6 and 423 CCSs; and WDXM2 37 °C, 3 and 725 CCSs. (*E*) Reconstructions of example STEM tomograms of clathrin structures in unroofed WDXM2 cells incubated at either RT or 37 °C. (*F*) Tracings of reconstructions overlaid each other. *n* = RT, 6; 37 °C, 8. (Scale bars, *A*, 500 nm; *C*, 200 nm; *F*, 50 nm.)

In mammalian and yeast CME, the endocytic proteins which are localized at the rim of the CME events are implicated in membrane bending, for example, Eps15/Ede1, Epsin, and FCHo/Syp1 (17–20). Therefore, based on the spatial and temporal profile of TPLATE and the ability of distinct domains of TPC to bind directly to the PM (21, 22), we hypothesize that components of the TPC are critical for membrane bending during plant CME. To test this notion, we looked directly at CCVs in plant cells subjected to TPC disruption. For these studies, we used the inducible TPLATE loss-of-function mutant WDXM2 in which *tplate* mutant plants are complemented with a genetically destabilized version of TPLATE that after heat shock results in the total aggregation of TPLATE away from the PM and blocks CME (23). First, we further confirmed that heat shock treatment had no significant effect on the efficiency and kinetics of CME in plants by examining fn4-64 uptake and the dynamics of single CME events (*SI Appendix*, Fig. S3). To achieve the spatial resolution required to look at the shape of individual CCVs, we used scanning electron microscopy (SEM) to examine metal replicas of unroofed protoplasts made directly from *wild-type* or *WDXM2* roots. Under control conditions in *WDXM2* cells, we observed that the majority of clathrin structures were spherical (i.e., fully invaginated CCVs). In contrast, in cells in which the TPC was disrupted, we observed many flat clathrin structures (Fig. 3*A*), which were never observed in control conditions. To quantify the effect of TPC disruption, we determined the shape of the clathrin structures by measuring the area and average intensity (a proxy for CCV curvature [*SI Appendix*, Fig. S4*A*]) (24) of each clathrin structure visualized and classified these shapes into four categories: “small and round” (the fully invaginated CCVs), “small and flat” (where curvature generation had failed), “large and round,” and “large and flat” (clathrin plaques) (Fig. 3 *B* and *C*). We found that the heat shock had no effect upon CCV formation in wild-type cells (*SI Appendix*, Fig. S4*B* and Fig. 3*D*), where the majority of clathrin structures were found as “small and round” (87 to 96%) (*SI Appendix*, Fig. S4*C*). This population in *WDXM2* cells under control conditions was 86%, but following TPC disruption, this decreased to 24%, and the “small and flat” population increased to 58% (compared to <7% in all other tested conditions). These results indicated that the TPC is required to generate curved clathrin structures.

To further confirm this, we directly examined the 3D shape of clathrin structures in *WDXM2* cells incubated at either control or TPC disruptive conditions using scanning TEM (STEM) tomography (Fig. 3*E*). Under conditions that disrupted TPC function, the curvature of clathrin structures did not exceed 10 nm, whereas under control conditions, the clathrin structures were spherical with Z heights >50 nm (Fig. 3*F* and Movies S2 and S3). Together, these ultrastructural examinations of clathrin structures demonstrate that the TPC is required to generate spherical CCVs.

### The TPLATE Complex Contains Domains Which Have Intrinsic Membrane Remodeling Activity.

**Fig. 4.**
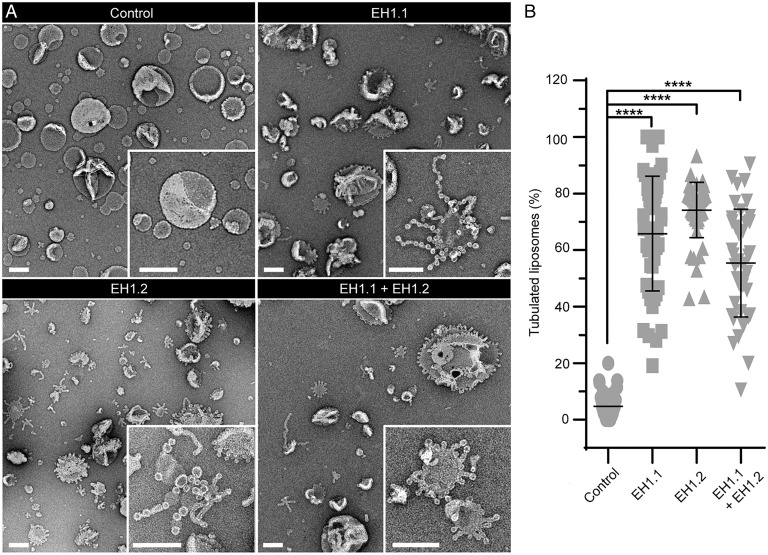
The AtEH1/Pan1 EH domains have membrane-bending activity. (*A*) Example TEM overviews of LUVs after 2 min incubation in control conditions or with EH domain EH1.1, EH1.2, and EH1.1 plus EH1.2. (*Insets*) Zooms of representative LUVs. (Scale bars, 200 nm.) (*B*) Quantification of the percentage of LUVs which displayed tubulation. N, control, 40; EH1.1, 47; EH1.2, 52; and EH1.1+EH1.2, 40 images pooled from three independent experiments. Plot, mean ± SD, ****P < 0.001, one-way ANOVA with Dunnett posttest to compare to control.

Given this strong phenotype of flattened clathrin structures during TPLATE disruption, we looked for protein domains within the TPC which could mediate membrane bending. The plant-specific members of the TPC, AtEH1/Pan1 and AtEH2/Pan1, each contain two Eps15 homology (EH) domains, which are also present in proteins which localize at the rim of CME events and are known to have membrane-bending activity in other systems (e.g., Eps15/Ede1 and Intersectin/Pan1) (13, 18–20). As the EH domains of Eps15 have been shown to tubulate membranes in vitro (20), and the EH domains of AtEH1/Pan1 have been shown to bind membranes (21), we therefore tested their ability to bend membranes. To do this, we incubated large unilamellar liposomes (LUVs) with the purified EH domains of AtEH1/Pan1, and analysis by TEM revealed that after 2 and 30 min, both EH domains produced significant levels of membrane ruffling and often long tubules of vesiculated membrane compared to control treatments (Fig. 4 and *SI Appendix*, Fig. S5). This demonstrated that the TPC, specifically AtEH1/Pan1, has the capacity to contribute to membrane bending.

## Discussion

The details of how endocytosis functions in plants, specifically how membranes bend to create endocytic vesicles against the extreme intracellular turgor pressure of plant cells, has been the biggest mystery in the field. This was further compounded by the fact that plant endocytosis does not rely upon the actin cytoskeleton to provide the force required to bend the endocytic membrane bending (8) and has been used previously as a main argument against the existence of efficient endocytosis in plants. Here, we identified a piece of this mechanism, showing that the plant-specific TPC serves as a critical mediator of membrane bending during plant endocytosis (*SI Appendix*, Fig. S6).

### Refinement of the TPLATE Complex Localization.

While the TPC is critical to plant CME, its precise localization and role during CME has remained elusive. Based on domain homology and biochemical interaction analysis, it had been predicted to localize under the clathrin coat functioning as a classical adaptor (9, 13). However, here we applied multiple approaches to study the fine dynamics and localization of the TPC at CME events, using state-of-the-art live imaging approaches to increase both the temporal and spatial resolutions to visualize live CME events and a biochemical analysis of purified plant CCVs to directly examine proteins encapsulated within the clathrin coat. Data from these different approaches together strongly suggest that TPLATE in fact localizes outside of the CCV.

First, by using a microscope-cooling stage to slow down the intracellular, trafficking processes (10), we were able to robustly visualize the order of dissociation of proteins from the CCV once freed from the PM at a much higher resolution than before (Fig. 1 *A*–*C*). In ∼28% of CCV departure traces, we found that the TPLATE and clathrin traces terminated at the same time, which could suggest that there is a fraction of TPLATE that is still bound to the PM invaginated during CME within CCVs or that even with the increased temporal resolution, we are unable to track the complete fate of every CCV before they leave the illumination volume. Furthermore, there is a small population of events in which clathrin dissociated from the CCV before TPLATE (∼6%), which could represent failed CME events. However, the significant majority of events demonstrated that TPLATE dissociated before clathrin, indicating that most of TPLATE at CME events is not trapped within the CCV coat. Secondly, the purification of CCVs from plant cells allowed us to specifically probe intact CCVs for endocytic proteins, which indicates if a protein is structurally incorporated within CCVs. Our Western blotting approach showed that while we detected a small fraction of TPLATE in the CCV fraction, there was no enrichment compared to background levels, which was the opposite trend for bona fide core CCV components like clathrin and AP2, which were greatly enriched in CCVs (Fig. 1 *D* and *E*). Notably, this result has been confirmed by mass spectrometry of purified CCVs, in which it was found that all members of the TCP failed to show any strong enrichment in CCVs (15). Thirdly, to increase our spatial resolution of live CME events, we used SIM. SIM provides a doubling of the optical lateral resolution compared to previous studies examining TPLATE [>200 to ∼100 nm (16)], and thus, we were able examine CME in intact plants at its highest resolution to date. We found that TPLATE, and other TPC members, presented on the PM as both foci and ring structures, which were rarer in occurrence (Fig. 2 and *SI Appendix*, Fig. S2). Upon examining the colocalization of TPLATE with CME events, we found that in a proportion (∼20%) of CME events, TPLATE is excluded from the clathrin assembly area during CME, which supports the findings from our other dissociation and biochemical analysis (Fig. 1). While we see this localization in ∼20% of CME events and that the ring is only present during some of the TPLATE lifetime on the membrane (Fig. 1*C* and Movie S1), this is likely because the resolution of SIM (∼100 nm) is greater than the average diameter of a spherical vesicle (∼80 nm). Therefore, the rings can only be resolved when the CME event is closer to 100 nm, which would represent the transient “dome” phase of endocytosis (2). However, because of this, it allows us to further predict that TPLATE is localized to the rim of the CME event and not within the clathrin coat assemble area (*SI Appendix*, Fig. S6*A*). By using 3D SIM, which has a Z resolution of ∼350 nm compared to ∼100 nm in TIRF-SIM (16), we still observed TPLATE ring formations and have enough Z resolution to be sure we are visualizing the whole endocytic invagination, which further supports the rim localization of TPLATE.

Thus, while we find a fraction of TPLATE which remains associated with CCVs and localizes with CME proteins in resolution-limited foci on the PM (which could be functioning as previously predicted), our advanced live imaging methodologies and direct biochemical analysis of purified CCVs suggest that the TPC is preferentially localized outside the CCV, at the rim of CME events.

### The TPLATE Complex Mediates Membrane Bending.

As the TPLATE localization outside of the CME event is more fitting of endocytic membrane bending machinery, we used electron microscopy to directly examine the effect of TPC disruption upon the formation of CCVs in vivo. We found that during TPC disruption, clathrin structures are flat instead of spherical, indicating a failure in membrane bending, thus directly implicating the TPC as a mediator of membrane bending (Fig. 3). How the TPC mediates membrane bending could either be by directly remodeling membranes itself or by acting as a hub recruiting other membrane-bending proteins. The idea that the TPC itself could directly have a role in membrane bending is given credence by the fact that the TPC contains several EH domains, homologous to those within the mammalian membrane bender Eps15 (∼35% sequence similarity between the plant and mammalian domains). Thus, to test if the TPC itself could be involved in membrane remodeling, we purified the EH domains of the TPC member AtEH1/Pan1 and showed that they have intrinsic membrane-remodeling activity in vitro, therefore demonstrating that the TPC possess membrane-bending machinery. However, how this activity could be mediated in vivo remains an open question, as AtEH1/Pan1 remains associated with the PM following TPC disruption (23) but is not sufficient to generate the invagination (Fig. 3). This separation of AtEH1/Pan1 and TPLATE, which in physiological conditions share over 90% colocalization and have identical dynamics on the PM (10), show that during *WDXM2* heat shock, the TPC losses its integrity, and the AtEH1/Pan1 interaction is lost, which then preferentially associates with its PM interaction partners (21, 23). While AtEH1/Pan1 remains on the PM during TPC disruption, it suggests that additional factors are required to modulate the membrane-bending activity of the AtEH1/Pan1 EH domains. This is similar to how EPS15 requires cofactors in mammals (20, 25), in which the isolated EPS15 EH domains can deform membranes while the full-length protein requires the cofactor FCHo (20). Interestingly, the Eps15 and FCHo interaction is mediated by the µ-homology domain (μHD) domain within FCho (26), which is similar to the interactions within the TPC, as the TPC member TML contains a µHD domain which interacts with AtEH1/Pan1 (13, 21); thus, it is tempting to hypothesize that the intact TPC is required to drive membrane bending in vivo. In support of this possible TPC mediated membrane bending, the TPC homologous TSET complex in Dictyostelium lacks the AtEH/Pan1 proteins (11, 22), suggesting a functional divergence between the plant TPC and the Dictyostelium TSET complex in which CME is also mechanistically distinct; as in contrast to plant CME, it is coupled with actin (27). While we cannot rule out the possibility that the TPC disruption prevents the recruitment of other critical membrane-bending components, we identify that the TPC itself has membrane-bending components and is required for the generation of curvature of vesicles during CME.

Overall, we refine the role of the TPC in plant endocytosis and provide insights into the evolutionary unique mechanism of membrane bending against high turgor pressure in plants. We show that the TPC functions as a mediator of membrane bending at the rim of endocytosis events. The plant-specific member of the TPC, AtEH1/Pan1, possess domains which have membrane-bending activity, thus providing further evidence for the evolutionary distinct mechanism of how endocytosis operates in plants.

## Materials and Methods

### Plant Materials.

*Arabidopsis thaliana* accession codes for genes used in this study: AP2A1 (AT5G22770), CLC2 (AT2G40060), TPLATE (AT3G01780), and AtEH1/Pan1 (AT1G20760). Transgenic *Arabidopsis thaliana* plants used in this study were *tplate pLAT52p::TPLATE-GFP* × *pRPS5A::CLC2-tagRFP*, *pLAT52p::TPLATE-GFP* × *pRPS5A::AP2A1-TagRFP*, *p35S::TASH3-GFP*, *p35s::LOLITA-GFP*, *tml-1 pTML::TML-GFP* (9), and *tplate pLAT52::WDXM2-GFP* (23).

### Growth Conditions.

Plants are grown by plating seeds on to 1/2- Murashige-Skoog (MS) agar plates with 1% (weight/volume) sucrose, stratified for 2 to 3 d in the dark at 4 °C, and then transferred to growth rooms (21 °C, 16 h light, 8 h dark) and grown vertically for 4, 5, or 7 d depending on the type of experiment for which they are required. Details of these incubation periods are expanded in the following methods sections related to specific experiments..

### Dissociation Analysis of CCV-Associated Proteins.

Raw data from Wang et al. (10) was analyzed to determine the departure dynamics of the endocytosis proteins. Briefly, spinning-disk microscopy was conducted on 4-d old epidermal cells of etiolated hypocotyls were imaged with a Nikon Ti microscope equipped with a Ultraview spinning-disk system (PerkinElmer), a Plan Apo 100× 1.45 numerical aperture (NA) oil immersion objective and a CherryTemp system (Cherry Biotech) to apply the experimental temperature conditions at either room temperature (25 °C) or 12 °C. Time lapses were collected at a frame rate of one frame per 1.174 s. The 12 °C time lapses of TPLATE-GFP and CLC2-TagRFP samples were subjected to histogram-matching bleach correction and then dynamically resliced to produce kymographs in Fiji (28). The CLC2 channel was manually screened to identify kymograph traces with a visible departure track. These selected traces were then examined to compare the departure of both channels and categized as illustrated in *SI Appendix*, Fig. S1.

### Western Blotting Analysis of CCV Purification.

CCVs were purified from suspension-cultured *Arabidopsis* T87W cells, as previously described (29). Equal amounts of protein from the deuterium ficoll gradient load (DFGL) and purified CCV samples were separated by sodium dodecyl sulphate–polyacrylamide gel electrophoresis (SDS-PAGE), transferred to nitrocellulose membrane, and immunoblotted with anti-CLC2 1:10,000 (30), anti-CHC 1:1,000 (sc-57684, Santa Cruz Biotechnology), anti-AP2mu2 1:250 (31), anti-TPLATE 1:2,000 (32), and anti-DRP1c 1:500 (33) antibodies. Primary antibodies were detected through anti-rabbit or anti-mouse secondary antibodies (Sigma-Aldrich) conjugated to horseradish peroxidase at 1:5,000 before application of SuperSignal West Femto enhanced chemiluminescent substrate (Thermo Fisher) and subsequent imaging with iBright CL1000 Imaging System (Thermo Fisher Scientific). The integrated density values of the chemiluminescent bands of the DFGL and CCV fractions were measured by ImageJ (NIH). The integrated density value of the CCV band was divided by the corresponding value of the DFGL band to determine the relative enrichment across three independent CCV purifications.

### FM Uptake and TIRF-M Imaging and Analysis.

A Zeiss LSM-800 confocal microscope was to examine the effect of fn4-64 uptake in 5-d-old Col-0 seedlings. Seedlings were incubated for 6 h at either room temperature or 35 °C for 6 h and then incubated with 2 µM fn4-64 in AM+ media for 5 min, washed twice in 1/2 Murashige and Skoog (MS) and 1% sucrose media, and imaged and analyzed, as specified previously (34). A 40× water immersion objected was used.

TIRF-M experiments made use of an Olympus IX83 inverted microscope equipped with a Cell^TIRF module using an OLYMPUS Uapo N 100×/1.49 Oil TIRF objective. For 6 h prior to imaging, 7-d old seedlings were incubated at either 25 or 37 °C. Root epidermal cells were imaged and analyzed as described previously (34); this provided unbiased lifetimes, densities, and fluorescence profiles of endocytosis proteins in samples subjected to the experimental temperature conditions.

### Super-Resolution Imaging of Endocytosis Events.

SIM was conducted on 7-d-old seedlings expressing TPLATE-GFP or AP2A1-GFP and CLC2-TagRFP at physiological temperature. Root samples were prepared as described previously (34), but high-precision 1.5 coverslips were used (Thorlabs, No. CG15CH), and epidermal cells in the elongation zone were selected for imaging. For 3D SIM, an OMX BLAZE v4 SIM (Applied Precision) was used. For TIRF-SIM, an OMX SR (GE Healthcare) was used. Both are equipped with a 60 × 1.42 NA oil immersion objective, and 100-mw, 488-nm, and 561-nm lasers were used for illumination (for TPLATE-GFP × CLC2-TagRFP, 488 laser powers ranged from 488, 30 to 100%; 561, 25 to 100%. For TPLATE-GFP × AP2A1-TagRFP, laser powers ranged from 488, 20 to 100%; 561, 40 to 100%). Images were reconstructed using SOFTWORX (GE Healthcare) and further processed in Fiji (28).

The colocalization rate was determined by using ComDet (https://github.com/ekatrukha/ComDet) in which colocalization was determined positive if spot detection was less than 4 pixels apart. This method uses wavelet decomposition to determine spot detection and thus considers rings and spots extremely close together as a single spot. To determine the pattern of localization of TPLATE, that is, if it is a spot or surrounding the CME event, spots were manually examined and scored if TPLATE presented as a crescent or ring around a CLC2 or AP2A1 spot.

### Ultrastructural Examination of CCVs by SEM and STEM Tomography from Metal Replicas of Protoplasts Made Directly from Roots.

Densely sown Col-0 or WDXM2-GFP plants were grown for 8 to 10 d. The roots were cut into small ∼1- to 2-mm fragments directly into “Enzyme solution” (0.4 M Mannitol, 20 mM KCl, 20 mM 2-(N-morpholino)ethanesulfonic acid (MES) pH 5.7, 1.5% Cellulase R10 [Yakult], and 0.4% Macerozyme R10 [Yakult] in H_2_0). The cuttings and enzyme solution were placed into a vacuum chamber for 20 mins and then subjected to a 3-h incubation at room temperature in the dark and with gentle agitation. The cells were then centrifuged at 100 rcf for 2 mins, and the pellet was washed with “W5 buffer” (154 mM NaCl, 125 mM CaCl_2_, 5 mM KCl, and 2mM MES) by centrifugation (100 rcf for 2 mins). The cells were then resuspended in W5 buffer and incubated at 4 °C for 30 mins. The sample was again centrifuged at 100 rcf for 2 mins, and the cells were resuspended in “hyperosmotic growth media (GM) buffer” (GM; 0.44% [wild type/volume] MS powder with vitamins [Duchefa Biochemie], 89 mM sucrose, and 75 mM mannitol, pH 5.5 adjusted with KOH) and then plated on precleaned (washed in pure ethanol and sonicated) carbon- (10 nm thickness) and poly-l-lysine- (Sigma) coated coverslips. Samples were incubated at room temperature in the dark for 30 min and then subjected to a 4-h incubation in the dark at either room temperature or 37 °C. Samples were then unroofed as described previously (34), with the buffers equilibrated to either room temperature or 37 °C. Samples for SEM analysis were attached to SEM mounts using sticky carbon tape and coated with platinum to a thickness of 3 nm, whereas samples for STEM were attached to a sticky Post-It note (as described in ref. 35) and coated with 3 nm platinum and 4 nm carbon using an ACE600 coating device (Leica Microsystems). The STEM samples were then washed with Buffered Oxide Etchant (diluted 6:1 with surfactant) to separate the metal replica from the coverslip, washed with distilled water, and remounted on formvar/carbon-coated 200-line bar electron microscopy grids (Science Services).

The SEM samples were then imaged with an FE-SEM Merlin Compact VP (Zeiss) and imaged with an In-lens Duo detector (in scanning electron mode) at an accelerating voltage of 3 to 5 kV. The area and mean gray value of clathrin-coated structures (CCSs) was measured using Fiji (28) in which regions of interest (ROIs) were manually drawn around each CCS. To estimate the curvature of the CCSs, the mean CCS ROI was divided by the average gray value of the PM (as determined by the mean gray value of 4 PM ROIs in each corner of each image). From these two values, the morphology of the CCS could be determined by using thresholds to divide the CCSs into categories as described by Moulay et al. (24). We used an area threshold of 8,500 mm^2^ (which is derived from a diameter of 105 nm) to determine if the CCS was small or large and a curvature value of 1.25 (determined by measuring the mean gray value of the large CCSs observed in TPC disruption conditions) to determine if the CCS was round or flat. Pooled data from multiple experiments were plotted, and the percentage of CCVs in each category was calculated.

STEM tomograms were recorded using a JEOL JEM2800 scanning/transmission electron microscope (200 kV). Each CCV was imaged over a range of −72 to 72°, with 4° steps driven by STEM Meister (https://temography.com/en/). Tomograms were then processed, and 3D reconstructions were made using Composer and Evo-viewer (https://temography.com/en/). To examine the curvature, 3D reconstructions were rotated 90°, and their profiles were manually traced in Adobe Illustrator.

### Expression and Purification of AtEH/Pan1 EH Domains.

The two EH domains of atEH1, EH1.1 and EH1.2 as defined by ref. 21, were amplified from synthetic AtEH1/Pan1 (codon optimized for bacterial expression, IDT) (*SI Appendix*, Table S1) and inserted into pET-TwinStrep-TEV-G4. They were then expressed in *Escherichia coli* BL21 cells and grown at 37 °C in lysogeny broth medium (pH 7.0) supplemented with 50 µg ml-1 kanamycin. Protein expression was induced at an optical density (OD600) of 0.6 with 1 mM isopropyl-β-thiogalactopyranoside and incubated for 5 h at 37 °C. Cultures were centrifuged at 5,000 *g* for 30 min at 4 °C, and pellets were resuspended in 50 mL phosphate-buffered saline buffer. They were then centrifuged at 4,700 *g* for 30 min at 4 °C, and then pellets were frozen and stored at −80 °C until further processing.

The pellets were resuspended for 1 h at 4 °C with gentle mixing in buffer A (20 mM Hepes [pH 7.4], 150 mM NaCl, and 2 mM CaCl2, as described by ref. 21) with supplemented ethylenediaminetetraacetic acid–free protease inhibitor mixture tablets (Roche Diagnostics), 1 mM phenylmethylsulfonyl fluoride, 1 mg mL-1 lysozyme, and 1 µg ml-1 deoxyribonuclease (DNase) I. Cells were lysed by sonication (Qsonica Q700) and centrifuged at 67,000 *g* for 1 h at 4 °C. The clarified lysate was incubated with Streptactin Sepharose resin (Strep-Tactin Sepharose resin; iba) for 1 hour at 4 °C. The resin was washed with 40 bed volumes of buffer A, and the fusion protein was eluted with buffer A containing 5 mM d-Desthiobiotin (Sigma-Aldrich). Peak atEH domain fractions were dialyzed overnight at 4 °C against buffer A in the presence of TwinStrep-tagged TEV protease (36) at a protease-to-sample molar ratio of 1:100. After centrifugation (21,140 × *g* for 10 min at 4 °C), the supernatant was applied to a HiLoad 16/600 Superdex 75 pg column, pre-equilibrated with buffer A, using a fast protein liquid chromatography system. Protein was eluted with buffer A and stored in aliquots at −80 °C. The protein sequences of the EH domains were verified by MS analysis.

### LUV Tubulation Assay.

LUVs were prepared using a mixture of 1,2-dioleoyl-sn-glycero-3-phospho-(1'-rac-glycerol), 1,2-dioleoyl-sn-glycero-3-phospho-L-serine, cholesterol (plant derived), and 1,2-dioleoyl-sn-glycero-3-phospho-(1'-myo-inositol-4',5′-bisphosphate) (PI(4,5)P_2_) (Avanti) at a ratio of 60:17.5:20:2,5 mol%. Lipids were mixed in a glass vial at the desired ratio, blow dried with filtered N2 to form a thin homogeneous film, and kept under vacuum for 2 to 3 h. The lipid film was rehydrated in a swelling buffer (20 mM Hepes [pH 7.4], 150 mM NaCl) for 10 min at room temperature to a total lipid concentration of 2 mM. The mixture was vortexed rigorously, and the resulting dispersion of multilamellar vesicles was repeatedly freeze thawed (five to six times) in liquid N2. The mixture was extruded through a polycarbonate membrane with pore size 400 nm (LiposoFast Liposome Factory). LUVs were stored at 4 °C and used within 4 d. To assay the membrane-bending activity of proteins of interest upon the LUVs, 10 µM of the protein of interest was mixed with 0.5 mM of LUVs in swelling buffer and incubated for 2 or 30 min at room temperature. Control LUVs were diluted to a concentration of 0.5 mM in swelling buffer and incubated for 1 h at room temperature. A total of 20 μL experimental solutions were incubated on glow-discharged carbon-coated copper EM grids (300 mesh, EMS). Filter paper was used to remove any excess solution, and the EM samples were then washed three times with swelling buffer. They were then negatively stained with 2% uranyl acetate aqueous solution for 2 min and observed under a Tecnai 12 transmission electron microscope operated at 120 kV (Thermo Fisher Scientific). The number of tubulated and nontubulated liposomes was counted manually using Fiji (28) from multiple experiments.

## Supplementary Material

Supplementary File

Supplementary File

Supplementary File

Supplementary File

## Data Availability

All raw data generated in this study is available on Zenodo, with the DOI: 10.5281/zenodo.5747101.

## Change History

December 21, 2021: The author line and affiliations have been updated.
