# Supplementary material for: The TPLATE complex mediates membrane bending during plant clathrin–mediated endocytosis
Source: Proc Natl Acad Sci U S A. 2021 Dec 14;118(51):e2113046118. doi: 10.1073/pnas.2113046118 (PMC8691179; doi:10.1073/pnas.2113046118)
Supplement: Supplementary File [file pnas.2113046118.sapp.pdf]

Supplemental Information

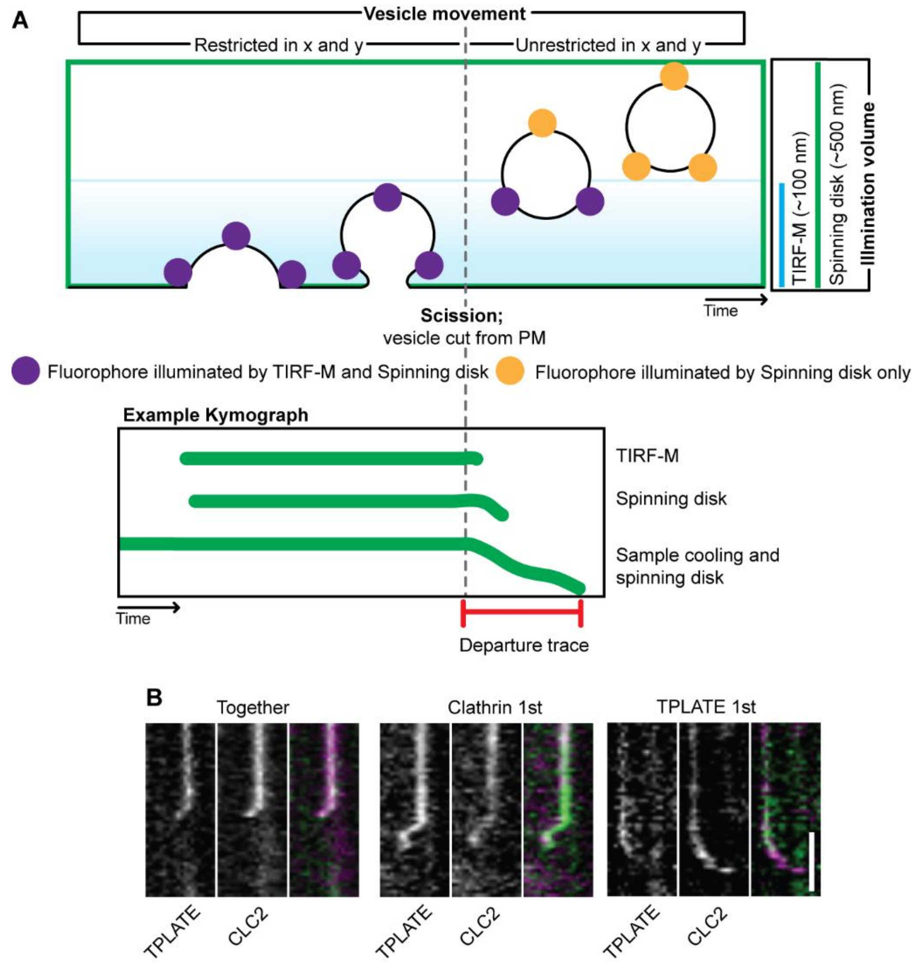

Fig. S1. Classification of departure events

Related to figure 1.

A) During CME, as the CCV invaginates on the PM it is restricted in movement in X and Y. This means in a kymograph it will present a linear pattern. However, once it is freed from the PM (gray dotted line), it trafficks away from the PM and thus is able to move in all directions, resulting in a lateral movement in the kymograph trace. Notably, this also results in the CCV moving deeper into the cell and exiting the illumination volume of fluorescence imaging methods, which determine how long the CCV is visible once after being cut from the PM and thus how long the departure trace is on a kymograph. TIRF-M has a Z depth of around 100-200 nm, whereas spinning disc has a Z volume of ~500 nm in Z away from the PM, which means fluorescently labeled CCVs are visible longer with spinning disk and thus have a longer departure trace. This is extended by using sample cooling to slow down cellular dynamics. B) Example departure traces of TPLATE (green) and CLC2 (magenta) at single events of CME demonstrating 4 different types of departure. 'Together', where both traces display the same lateral movement at the end of the trace and disappear together, which would be expected if TPLATE is bound to the CCV under CLC2 as it cannot departure the CCV before uncoating occurs before leaving the illumination volume; 'Clathrin 1st', where the CLC2 trace disappears before the TPLATE trace, which could indicate that CLC is removed as a layer before TPLATE

and ‘TPLATE 1st’, where the TPLATE trace terminates before the CLC2 trace, indicating that it is free to leave the CCV before the clathrin coat dissociates.

Scale bar, B, 60 s.

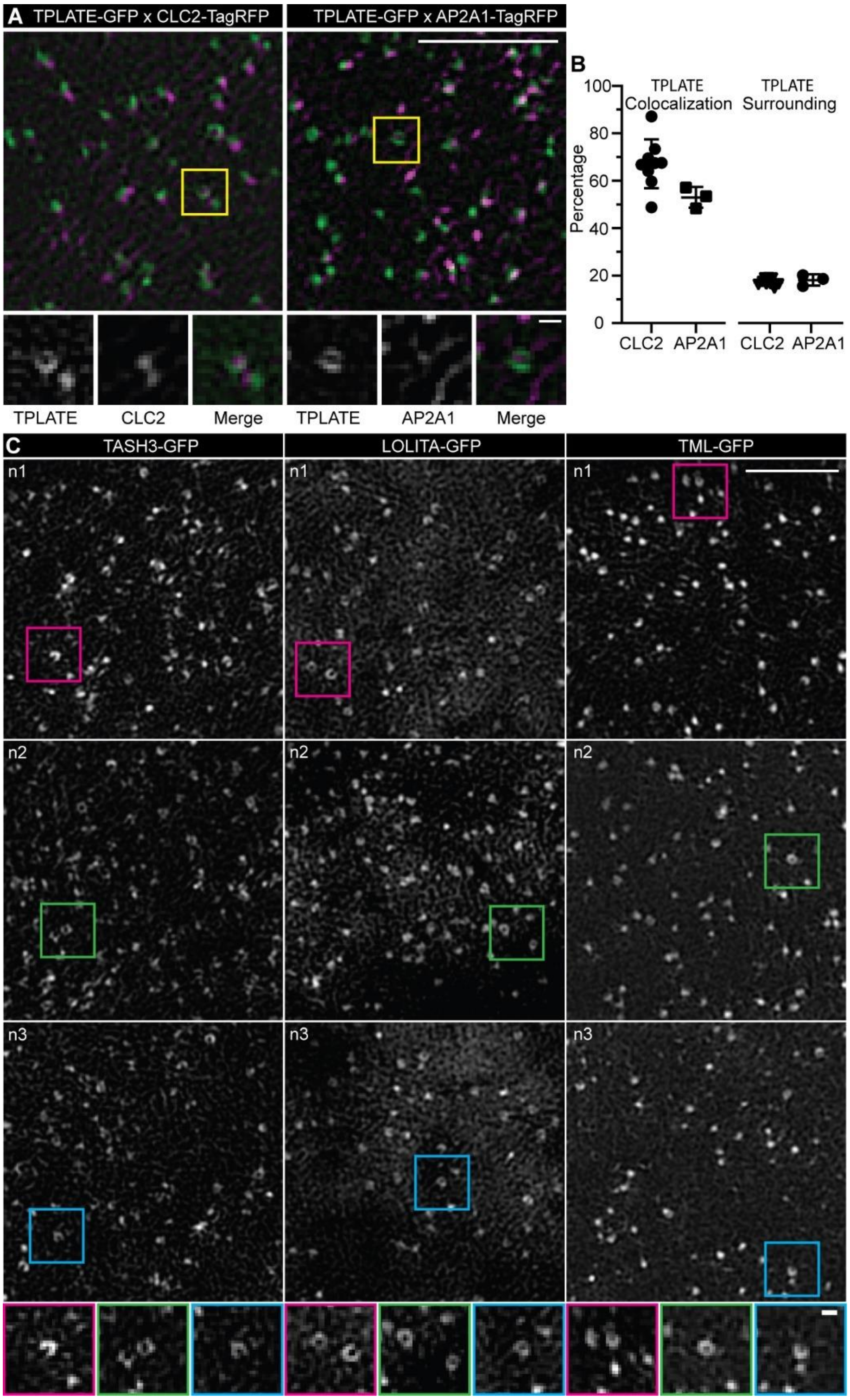

Fig. S2. TPLATE localizes around Clathrin and AP2

Related to figure 2.

A) TIRF-SIM example images of *Arabidopsis* root epidermal cells expressing A) TPLATE-GFP (green) x CLC2-TagRFP (magenta) and TPLATE-GFP x AP2A1-TagRFP. Yellow squares denote the area zoomed in for the lower panels. C) Quantification of colocalization of TIRF-SIM images of TPLATE with CLC2 and AP2A1, and the percentage of colocalized spots where TPLATE is found to surround CLC2 and AP2A1. Plots, mean ± SD. N; TPLATE x CLC2, 9 cells; TPLATE x AP2, 3 cells. C) TIRF-SIM images of epidermal cells from 3

independent (n1-n3) *Arabidopsis* root tips expressing additional TPC members (TASH3-GFP, Lolita-GFP and TML-GFP). Magenta, green, and blue squares denote areas zoomed in for the lower panels from the experimental repeats.

Scale bars, A and C, upper images, 2 µm, lower panels, 200 nm.

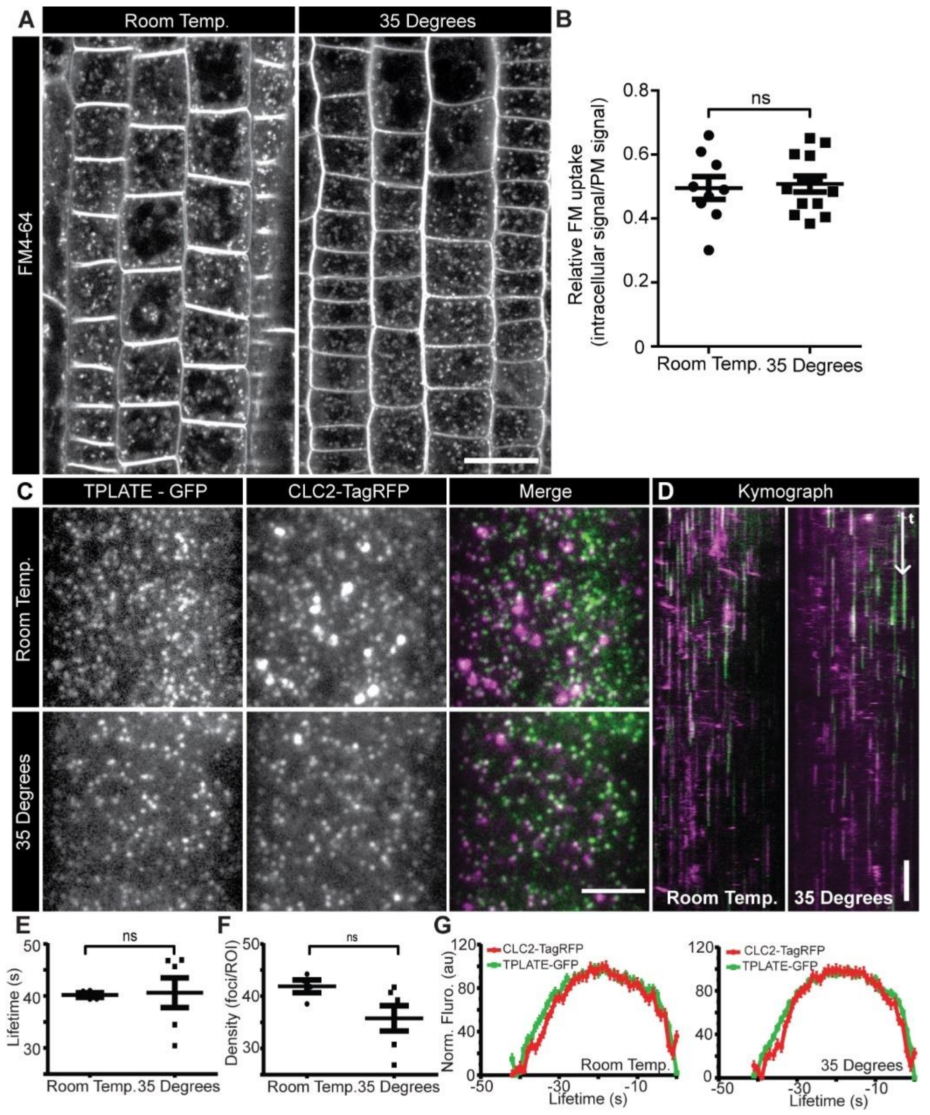

Fig. S3. Heat shock does not affect the efficiency and dynamics of endocytosis.

Related to figure 3.

A) Confocal images of Arabidopsis roots incubated with FM4-64 after a 6-hour incubation at room temperature or 35°C. B) Quantification of FM uptake from multiple experiments. N; room temperature, 9 independent seedlings, 169 cells; 35°C, 13 independent roots, 245 cells. C) Example TIRF-M images of Arabidopsis root epidermal cells expressing TPLATE-GFP (green) and CLC2-TagRFP (magenta) after incubation at room temperature or 35°C for 6 hours. D) Typical kymographs obtained from data shown in C. E) The lifetimes, F) density and G) mean profiles of endocytosis events combined for multiple experiments of seedlings incubated at room temperature or 35°C for 6 hours. N; room temperature, 4 independent roots, 18473 events; 35°C, 6 independent roots, 22223 events. Ns = not significant (P > 0.05) t-test result. Scale bars, A, 20 µm; C, 5 µm; D, 60 s.

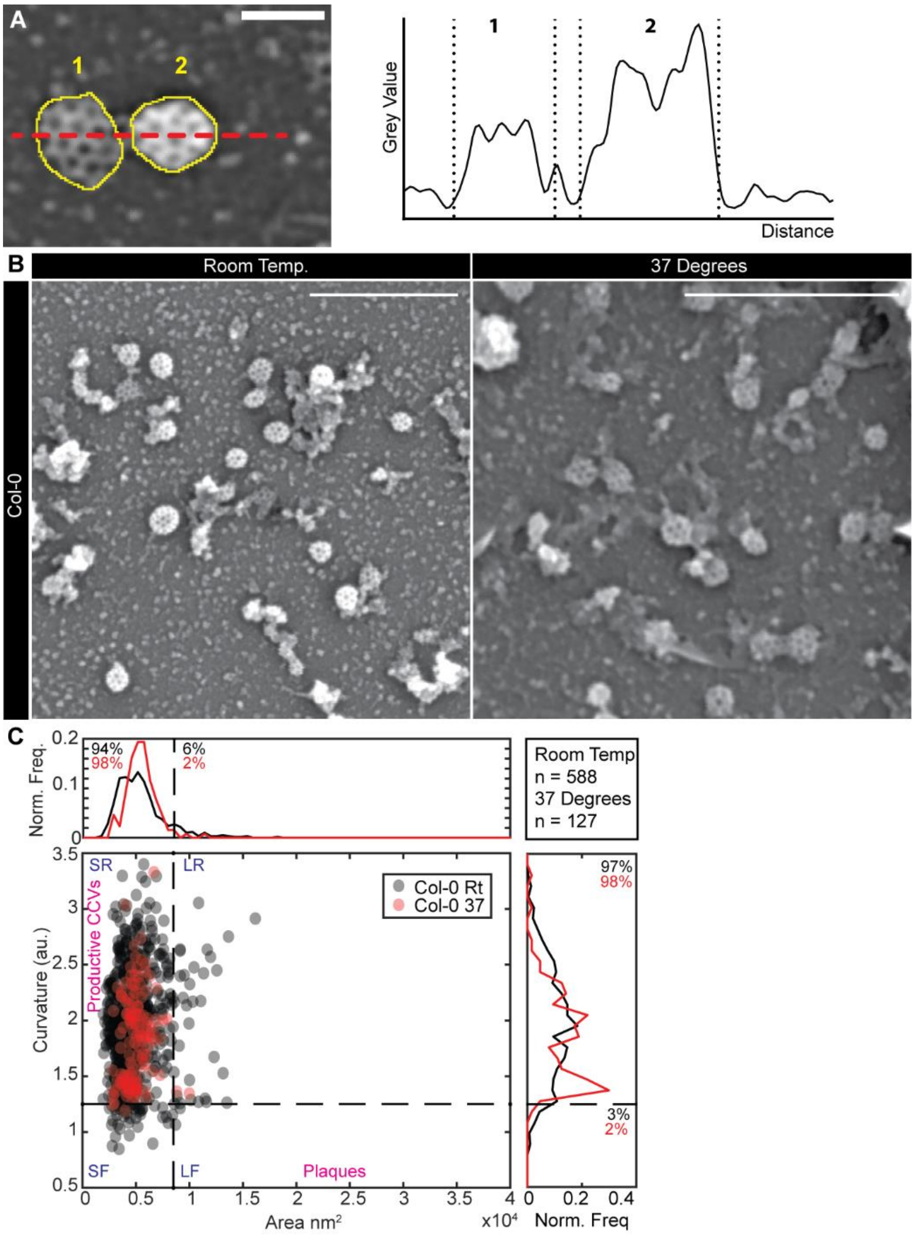

Fig. S4. Heat shock does not affect the formation of spherical clathrin-coated vesicles

Related to Figure 3.

A) ROIs (yellow dashed line) are drawn around the clathrin-coated vesicles (CCS) to determine the size and the mean gray values. The gray values can be used to estimate the curvature of the CCSs, for example, the flatter CCS (1) has a lower grey value than the rounder CCS (2). B) Representative SEM images of metal replicas of unroofed Col-0 root protoplast cells. C) Scatter plot of the area and curvature of CCSs in wild-type Col-0 cells incubated at room temperature (RT) (gray dots) or 37°C (red dots) for 4 hours and classified into 4 types of CCSs as detailed in the methods. Scale bars, 500 nm. N = RT, 3 and 588 CCSs; 37°C, 3 and 127 CCSs. Scale bars, A, 100 nm; B, 500 nm.

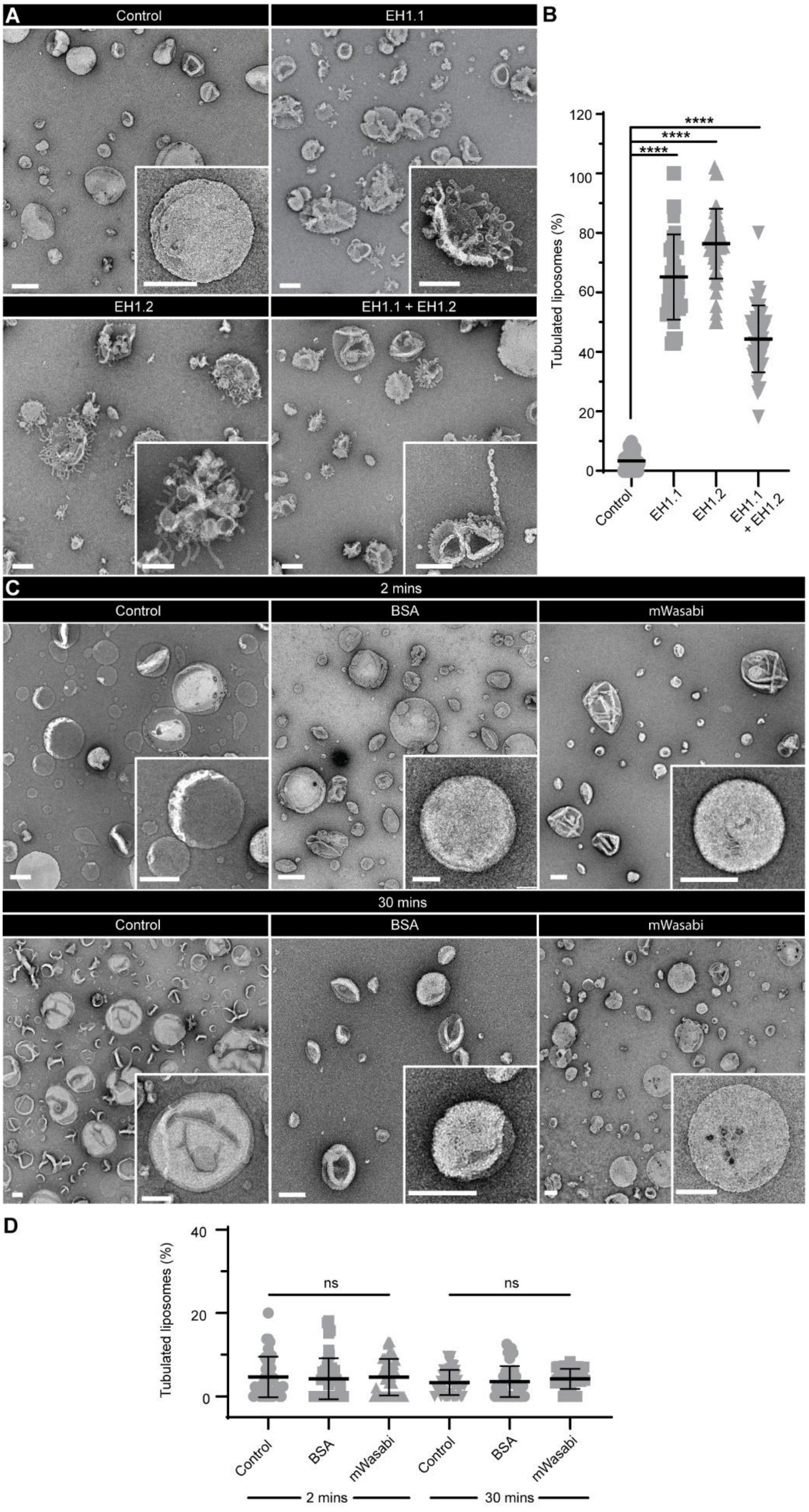

Fig. S5. AtEH1/Pan1 EH domain membrane bending activity and additional controls

Related to figure 4.

A) Example TEM overviews of LUVs after 30 minutes incubation in control conditions, or with EH domain EH1.1, EHD1.2 and EHD1.1 plus EHD1.2. Inserts are zooms of representative LUVs. B) Quantification of the percentage of LUVs which displayed tubulation. N; control, 41; EH1.1, 40; EH1.2, 44 and EH1.1+EH1.2, 46 images pooled from 3 independent experiments. C) Example TEM overviews of LUVs after 2 minutes (upper panels), or 30 mins (lower panels) incubation in control conditions, or with BSA, or mWasabi. Inserts are zooms of representative LUVs. D) Quantification of the percentage of LUVs which displayed tubulation. N; control 2 min, 41; BSA 2 min, 48; mWasabi 2 min, 42; control 30 min, 41; BSA 30 min, 41 and mWasabi 30 min, 40 images pooled from 3 independent experiments. Plot, mean ±SD. **** $p < 0.001$, one-way ANOVA with Dunnett post-test to compare to control, 'ns' indicate not significant.

Scale bars, 200 nm.

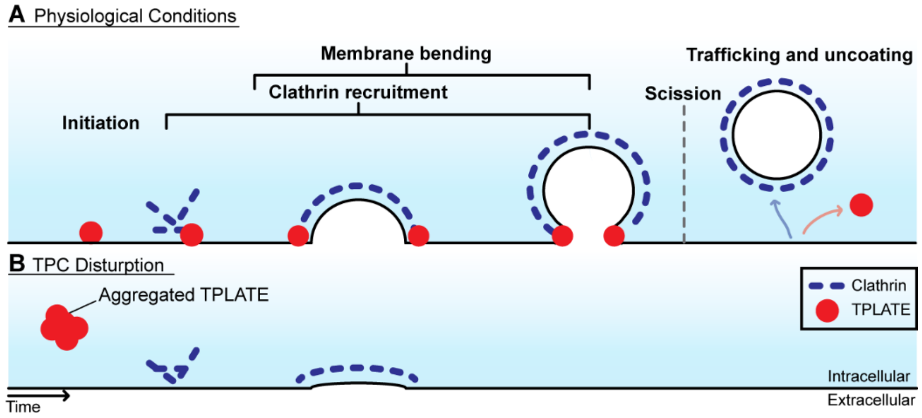

Fig. S6. The TPC is required for membrane bending during plant CME

A) In physiological conditions TPLATE and the TPC is recruited to the PM and clathrin is then recruited to begin the coat assembly phase of CME. Then the membrane bends creating an invagination against the high turgor pressure, here TPLATE is located at the rim of this invagination and drives the membrane bending. Eventually as the CME vesicle grows, a tight neck is created, where the TPC ring closes and after scission is able to departure the CCV before clathrin as it is not associated within the coat. B) During TPC disruption, TPLATE aggerates in the cytoplasm, while clathrin and AtEH1/Pan1 is still recruited to the PM but no invagination is created as there the full TPC is not present, resulting in the failure of membrane bending during the CME event.

Movie S1. TIRF-SIM of root epidermal cells expressing TPLATE-GFP

Example TIRF-SIM time lapse of TPLATE-GFP in an *Arabidopsis* root epidermal cell. Yellow ovals denote TPLATE structures which dynamically form ring structures. Time interval between frames, 5 seconds. Scale bar, 5 µm.

Movie S2. STEM tomography of a clathrin-coated vesicle in control conditions

180° rotation of a 3D reconstructed STEM tomogram for a CCV in a metal replica of an unroofed WDXM2 cell after 4-hour incubation at room temperature.

Movie S3. STEM tomography of a clathrin-coated vesicle during TPC disruption

180° rotation of a 3D reconstructed STEM tomogram for a CCV in a metal replica of an unroofed WDXM2 cell after 4-hour incubation at 37°C.

Construct	Sequence
*At*EH1/Pan1 EH1.1	GCGGGACAAAATCCAAACATGGATCAATTCGAGGCTTACTTTAAGC GCGCAGACCTCGATGGCGATGGCCGCATTTCGGGTGCTGAAGCCGT AGGATTCTTTCAGGGTTCAGGCCTCTCTAAGCAAGTCCTTGCCCAG ATTTGGAGTCTCAGTGATAGATCTCACTCCGGTTTCCTCGATCGCC AGAATTTCTATAACTCGTTGCGCCTCGTAACCGTAGCTCAATCGAA GAGAGATCTCACTCCTGAAATTGTCAATGCTGCGTTGAACACGCCT GCTGCAGCGAAAATCCCGCCTCCTAAGATAAATCTCAGCGCA
*At*EH1/Pan1 EH1.2	GGTAACAATCAGCCGCCTTGGCCCAAAATGAAACCGTCCGACGTAC AAAAATATACTAAAGTCTTCATGGAAGTTGACAGTGACAAGGACGG TAAAATAACTGGAGAACAGGCTCGTAATCTCTTTCTTTCGTGGCGC TTGCCTCGTGAAGTTTTGAAGCACGTGTGGGAGCTTTCTGATCAGG ACAACGACACCATGCTGAGTCTGCGTGAGTTCTGTATTTCTTTGTA CCTGATGGAGCGCTATCGCGAGGGAAGGCCATTGCCCACCGCATTG CCGAGCTCTATAATGTTTGATGAAACTCTTCTGTCG
pET_TST_EH1.1_fwd	GAAAACCTGTATTTTCAGGGCGCGGGACAAAATCCAAACATG
pET_TST_EH1.1_rev	TCGAGTGCGGCCGCAAGCCTATGCGCTGAGATTTATCTTAGGAG
pET_TST_EH1.2_fwd	GAAAACCTGTATTTTCAGGGCGGTAACAATCAGCCGCCTTG
pET_TST_EH1.2_rev	TCGAGTGCGGCCGCAAGCCTACGACAGAAGAGTTTCATCAAACATT ATAG

Table 1 – Codon optimized AtEH1/Pan1 EH domain sequences and primers used in this study
